# THE INFLUENCE OF SOCIETAL CRISES ON SOCIAL INTEGRATION

**DOI:** 10.1093/geroni/igae098.0602

**Published:** 2024-12-31

**Authors:** Lisa Klasen, Mareike Buenning, Nadiya Kelle, Oliver Huxhold

**Affiliations:** German Centre of Gerontology, Berlin, Berlin, Germany; German Centre of Gerontology, Berlin, Berlin, Germany; German Centre of Gerontology, Berlin, Berlin, Germany; German Centre of Gerontology, Berlin, Berlin, Germany

## Abstract

Societal crises have profound effects on various life domains. So far, the literature has focused on the effects of crises on financial security or health. Effects on social integration, i.e., social relationships and social participation, have received little attention, despite of their relevance for physical and mental health. The COVID-19 pandemic brought this topic to the forefront of the scientific and societal debate. However, to date no theoretical framework has been developed that captures the breadth and long-term influence of societal crisis on social integration. This presentation introduces a new theoretical framework – the DIRe-Crises-Model – for analyzing the effects of social crises on social integration, building on the Differential Investment of Resources (DIRe) Model. According to the DIRe-Crises-Model, crises influence social integration via four pathways by: a) limiting the time and energy that can be invested in social relationships, b) restricting the social opportunity structure, c) influencing individual characteristics essential for resource investment, and d) influencing the effects of social relationships (e.g., changes in the experience of loneliness). Dynamic components in the model allow to derive hypotheses on the long-term development of the different effects pathways of crises on social integration. Moreover, social inequalities can moderate the effects of the different pathways of crisis on social integration. The DIRe-Crises-Model as a theoretical framework has the potential to guide empirical analysis on the effects of any given societal crisis on social integration. As such, the framework may help to manage future crises, for example, by stimulating targeted interventions.

